# RNA helicase DDX5-induced circPHF14 promotes gastric cancer cell progression

**DOI:** 10.18632/aging.204623

**Published:** 2023-03-30

**Authors:** Jia Wang, Chunjie Han, Jinsheng Wang, Qiu Peng

**Affiliations:** 1 Hunan Key Laboratory of Cancer Metabolism, Hunan Cancer Hospital and The Affiliated Cancer Hospital of Xiangya School of Medicine, Central South University, Changsha 410013, Hunan, China; 2 Department of Immunology, Changzhi Medical College, Changzhi 046000, Shanxi, China; 3 Collaborative Innovation Center for Aging Mechanism Research and Transformation, Center for Healthy Aging, Changzhi Medical College, Changzhi 046000, Shanxi, China; 4 Department of Orthopaedics, Heji Hospital Affiliated to Changzhi Medical College, Changzhi 046000, Shanxi, China; 5 Key Laboratory of Esophageal Cancer Basic Research and Clinical Transformation, Heping Hospital Affiliated to Changzhi Medical College, Changzhi 046000, Shanxi, China; 6 Department of Pathology, Changzhi Medical College, Changzhi 046000, Shanxi, China

**Keywords:** gastric cancer, tumor

## Abstract

As a well-established member of a strongly conserved protein family, DDX5 binds to RNA helicase in a specific manner, which can regulate mRNA transcription, protein translation and synthesis and precursor messenger RNA processing or alternative splicing. The effects of DDX5 on carcinogenesis and cancer progression are increasingly evident. Circular RNAs (circRNAs), a novel group of functionally non-coding RNAs (ncRNAs) with disordered expression, are associated with various pathological processes (e.g., tumors). circRNA pattern and its function regulated by DDX5 have not yet been determined. According to our findings, DDX5 was dramatically upregulated for stomach cancer tissues, and its overexpression contributed to the cell growth and invasion of GC cells. Based on the analysis of genome-wide circRNAs conducted with circRNA sequencing, DDX5 induces a large number of circRNAs. Further to screen several circRNAs from PHF14 for function, it was found that circPHF14 was essential for the growth and tumorigenesis of DDX5-positive gastric cancer cells. These findings suggest that in addition to the messenger RNA and microRNA patterns, DDX5 also effects a circRNA pattern, as demonstrated by circPHF14. DDX5-induced circRNAs have been found to be of crucial importance for the growth of DDX5-positive gastric cancer cells, providing a new therapeutic target.

## INTRODUCTION

Stomach cancer is the most widespread malignancy in the digestive system. and its mortality ranks second among cancers in the world, with 7.83 million deaths in 2018 [1, 2]. Although with the continuous development of new diagnostic techniques, the five years survival ratio for gastric cancer is gradually increased, gastric cancer is still the main burden on a global scale, especially in Eastern Asia [3, 4]. The etiology and pathogenesis of gastric cancer are very comprehensive and related to a variety of reasons. H. pylori infection and dietary habits are recognized as a major factor contributing to the development of gastric cancer [5]. Surgical procedures are still the most successful treatment for stomach cancer [6]. The main challenges of advanced gastric cancer treatment are the manifestations of peritoneal, distant organ and lymphatic metastases [7]. Therefore, identifying key molecular functions in gastric carcinogenesis and progression will help develop new and effective therapeutic strategies.

DEAD (Asp-Glu-Ala-Asp) box RNA helicase 5 (DDX5) as an ATP-dependent RNA helicase that plays a key role in transcription initiation and RNA splicing. It is also a transcriptional co activator of a variety of tumor-related transcription factors, [8, 9]. As an oncogene, DDX5 is overexpressed in many tumours, promoting tumour cell growth and metastasis, including cancers of the breast, colon, prostate, non-small cell lung cancer (NSCLC) and glioma [10–13]. In addition, DDX5 can associate with β-catenin to form a complex, which promotes promote the expression of multiple oncogenes, like the cyclin D1, fra1, c-Myc, etc. [14]. DDX5 is often abnormally amplified in gastric cancer. Although there is increasing evidence that DDX5 influence the progression of gastric cancer [15], effect and functional role of DDX5 on gastric cancer remains to be further studied.

CircRNAs are a group of closed circular RNA molecules formed by the downstream 3’ and 5’ ends of the linear RNA sequence. Circular RNA lacks 3’ poly and 5’ cap (adenylate) tail structure, and it is stable and not easy to be degraded by nuclease [16]. CircRNAs have been found in many mammalian cells, which can participate in gene expression and post transcriptional expression regulation [17, 18]. Recently, increasing evidence indicates that circular RNA affects the development of certain diseases via modulating gene transcription, including cancer [19, 20]. Moreover, some investigations were conducted to find correlation between the expression levels of some cyclic RNAs in tumour cells and the extent of tumour metastasis [21–23]. New evidence suggests that circular RNA may represent an active and specific target for the diagnosis and treatment with gastric cancer [24–26]. Therefore, exploring the molecular mechanism of circRNAs may offer novel perspectives and approaches to the diagnosis, treatment and prognosis of gastric cancer patients.

Although DDX5 and circRNA serve a very important function regarding the progression on gastric cancer, it is unclear whether DDX5 can participate in gastric cancer progression through the induction in circRNA expression. In this work, we determine that DDX5 is expressed at elevated levels within gastric cancer tissues and identify the effects of DDX5 in enhancing the growth and invasion of gastric cancer cells *in vitro*. Moreover, we have analyzed RNA sequencing data of RNase R-producing treatments in AGS gastric cancer cells expressing DDX5 gene and an extensive range of DDX5-induced circRNAs were ascertained, of which circPHF14 promotes proliferation and carcinogenesis of DDX5-positive gastric cancer cells both *in vivo* and *in vitro*. We also found that DDX5 can induce the expression of a significant number of circular RNAs by RNase R-treated RNA sequencing was performed by over-expression of DDX5 in AGS cell, in which circRNAs circPHF14 accelerated the growth and tumorigenesis of DDX5-positive cells. Therefore, these results revealed that DDX5 and these DDX5-induced circRNAs are critical in the progression of gastric cancer and possible potential targets for drug therapy.

## MATERIALS AND METHODS

### Cell culture

The AGS and 7901 gastric cancer cells were cultured in RPMI-1640 medium containing 10% fetal bovine serum (FBS). Cell lines were obtained from American Type Culture Collection (https://www.ATCC.org, ATCC) for this study. A mycoplasma test was negative for the cell line.

### Plasmid construction, and transfection with short interfering RNA (siRNA)

DNA fragments encoding Flag-DDX5 was created by PCR with cloning into the p3×Flag-CMV-10 empty vector. All siRNAs in this study were obtained from Ribobio (Guangzhou, China). All siRNAs and plasmids in this study were transfected into cells by transfection reagent Lipofectamine 3000 (Invitrogen). CircPHF14 siRNA targeting sequences (5’- TCATCTTCCAAGAGAACCA -3’ or negative control sequences (5’-TTCTCCGAACGTGTCACGT-3’). CircPHF14 knockdown was identified by qPCR.

### Real-time quantitative PCR (qPCR)

2 μg of total RNA was reverse transcribed into cDNA using Reverse transcription kit (Vazyme, Nanjing, China). The qPCR kit was obtained from Takara. All primers in the present research were synthesized by Sangon (Shanghai, China). Human GAPDH or U6 GAPDH or U6 were used as negative controls. The expression of each genetic profile was quantified by measuring the cycle threshold, and the relative change of gene expression was calculated by mathematical method. Primer sequences are shown as follows: GAPDH, F-5’-GGGAGCCAAAAGGGTCAT-3’, R-5’- GAGTCCTTCCACGATACCAA-3’. CircPHF14, F-5’-GTGATTCTTCATCTTCCAAGAGAAC-3’, R-5’- TGCAACACAGACTGTCTTTGTCTTC-3’.

### Western blot

The proteins were extracted using RIPA lysate containing protease inhibitors, and the extracted proteins were quantified by BCA. 30 μg of protein was used for SDS-PAGE in each treatment group, and the proteins were transferred to PVDF (Millipore) and incubated sequentially with primary and secondary antibodies for the target proteins.

### Bioinformatics analysis

The analysis of Oncomine database (http://www.oncomine.org) was made as described before [27]. Shortly, we used the following threshold values to evaluate the expression of DDX5 in gastric cancer tissues and corresponding normal gastric tissues: fold-change of 2, P value of 0.05. The UALCAN database (http://ualcan.path.uab.edu/index.html) is used to analyze public TCGA samples. The Human Protein Atlas database (https://www.proteinatlas.org/) was used to obtain the immunohistochemical detection data of DDX5 in gastric cancer specimens and corresponding non-cancerous samples.

### RNase R and actinomycin D treatment

Actinomycin D (Sigma, USA) treated cells blocked RNA transcription to detect the stability of circPHF14 and linear PHF14 mRNA, and dimethyl sulfoxide (DMSO) was used as negative control. Take the final concentration of 1 μg/mL DMSO or actinomycin D treated cells for 0, 8, 16 and 24 h, RNA was extracted and detected by reverse transcription q-PCR. For the RNase R digestion, TRIzol was used to extract total RNAs using RNase R processing in buffer added with Ribonuclease Inhibitor at 37° C for 1 h. Reverse transcription Kit (Vazyme, Nanjing, China) reverse transcribed RNA with RNase R-treated to synthesize cDNA and detected by q-PCR.

### circRNA sequencing

Following the manufacturer’s instructions, total RNA was isolated using the RNeasy Mini kit (Qiagen). RNA quality was ensured by adding DNase I in column digestion. Two biologically replicated samples were conducted for each treatment. For circRNA sequencing, before the RNA library was constructed, total RNA was digested with RNase R at 37° C for 1 h. NEBNext® Ultra™ Directional RNA Library Prep Kit for Illumina (E7420L) was used to construct an RNA library for circular RNA sequencing. A paired-end sequencing experiment was performed at RiboBio Co., Ltd. or Amogene Biotech Co., Ltd. with Illumina HiSeq 3000. Under accession number GSE207636, the raw expression files and details are available in NCBI GEO.

### Cell proliferation assay

To analyze cell proliferation by Cell Counting Kit-8 (CCK-8; Biotool, China). Briefly, 96 well plates were used in each group, and 1000 cells were cultured in each well. From 1 day to 4 days after incubation, 10 ml of CCK-8 reagent was added to each of the well and placed at 37° C for 2 hours. Measuring the absorbance value of 450nm.

In the plate colony formation experiment, 1000 treated gastric cancer cells were cultured in 6-wellplates and cultured for two weeks in RPMI-1640 medium supplemented with 10% fetal bovine serum for two weeks. Then, the cells were cleaned with PBS, fixed with 4% paraformaldehyde, and most stained with Viola crystals. Microscope and Image J software were used to estimate the percentage and intensity of the area covered by stained cell colonies.

EdU incorporation assay was performed after 48 hours of culture of the treated cells based on the instruction manual. Then, the cells were washed and stained with buffer containing DAPI, and imaged were collected using a fluorescence microscopy.

### Cell migration assays

Transwell containing polycarbonate filters with 8-μm pores was used for cell migration testing. 5×10^5^ cells were suspended in serum-free medium and added to the upper chamber; Add RPMI-1640 medium (10% fetal bovine serum) to the small chamber underneath. The migrated cells were cleaned with PBS, fixed in 4% paraformaldehyde and finally stained with Viola crystal.

### Immunofluorescence

Cells were fixed using formalin, then the cell membrane was permeabilized with Triton X-100, incubated with DDX5 antibody and circPHF14 probe followed by fluorescent secondary antibody, and finally photographed with fluorescent microscope.

### Animal experiment

All animal euthanasia and care protocols were approved by the Institutional Animal Care and Use Committee of Changzhi Medical College (Changzhi, China). In the research of cancer cell xenograft, about 3-4 weeks old nude mice were injected subcutaneously 5×10^6^ AGS cells in the right abdomen of the mice transfected with DDX5/ DDX5+si-circPHF14/negative control (nc) in 100 μl serum-free medium that was mixed with matrigel (1:1). The mice were dissected for about 4 weeks, and the tumor tissues were taken out for fixation, dehydration and photography.

### Statistical analysis

Statistical analysis was conducted utilizing Graphpad prism 8 and SPSS 19.0 software. Data were typically expressed as the mean ± SD, and the differences between groups were assessed with the unpaired Student t-test. P < 0.05 was regarded as be significant.

### Availability of data and materials

The datasets, cell lines, plasmids and other reagents described in this manuscript are available upon a reasonable request.

## RESULTS

### DDX5 expression is correlated with poor patient survival in gastric cancer

**Figure 1:**
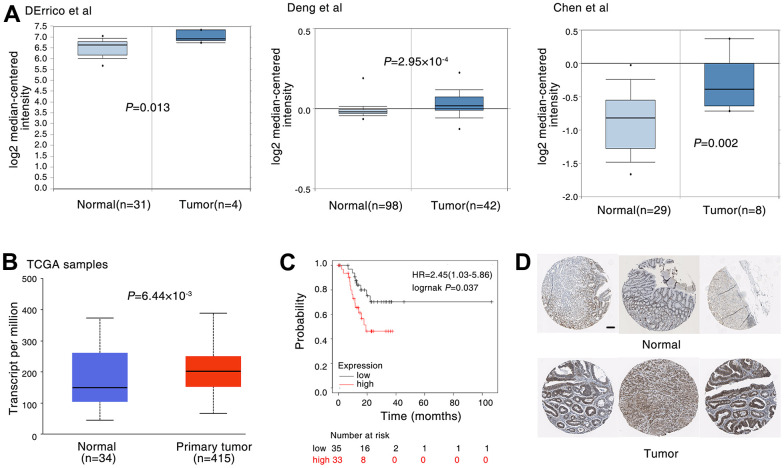
**DDX5 expression is correlated with poor patient survival in gastric cancer.** (**A**) The expression of DDX5 was analyzed in patients with gastric. DDX5 expression in gastric cancer samples and non-cancer controls; the publicly accessible gene expression data of DDX5 was obtained from Oncomine database. (**B**) DDX5 expression in gastric cancer samples and non-cancer controls; The publicly accessible gene expression data of DDX5 was obtained from The Cancer Genome Atlas (TCGA) database. (**C**) Kaplan-Meier overall survival curves according to DDX5 expression in patient cohorts in KM-plotter database. The percentage of survival patients in high DDX5 and low DDX5 groups at different time points are presented. (**D**) DDX5 expression in gastric cancer patient with or non-cancer controls was measured by immunohistochemical staining. The publicly accessible protein expression data of DDX5 was obtained from The Human Protein Atlas database. Data were analyzed with Student’s t-test, p values were shown. Scale bar 200μm.

To further characterise the connection of DDX5 and gastric cancer, we analysed its expression in the gastric tissues of tumour patients. Oncomine database is used to obtain the public gene expression data of DDX5 [28]. We analyzed three datasets from DErrico et al., Deng et al., Chen et al. [29–31]. And a significantly higher DDX5 expression in tumour tissues compared to non-cancerous normal tissues. (Figure 1A). Through The Cancer Genome Atlas database [32], we validated that DDX5 expression was significantly higher in gastric cancer samples than in non-tumour controls. (Figure 1B). Using the gene expression dataset of gastric cancer patients from the KM Mapper database, an association study between DDX5 and overall survival was conducted. Patients with higher DDX5 expression were found to have a lower overall survival when compared to those with low DDX5 expression (Figure 1C). Next, the expression of DDX5 in normal tissues and gastric cancer patients was analysed in The Human Protein Atlas database. In agreement with the results from public databases, DDX5 expression was higher in gastric cancer specimens than in normal specimens. (Figure 1D). We also further examined the protein expression levels of DDX5 in multiple cell lines by western blot assay and found that the protein expression levels of DDX5 were generally higher in gastric cancer cells than in normal cells GES1 (Supplementary Figure 1).

### DDX5 promotes cancer cell growth and migration

**Figure 2:**
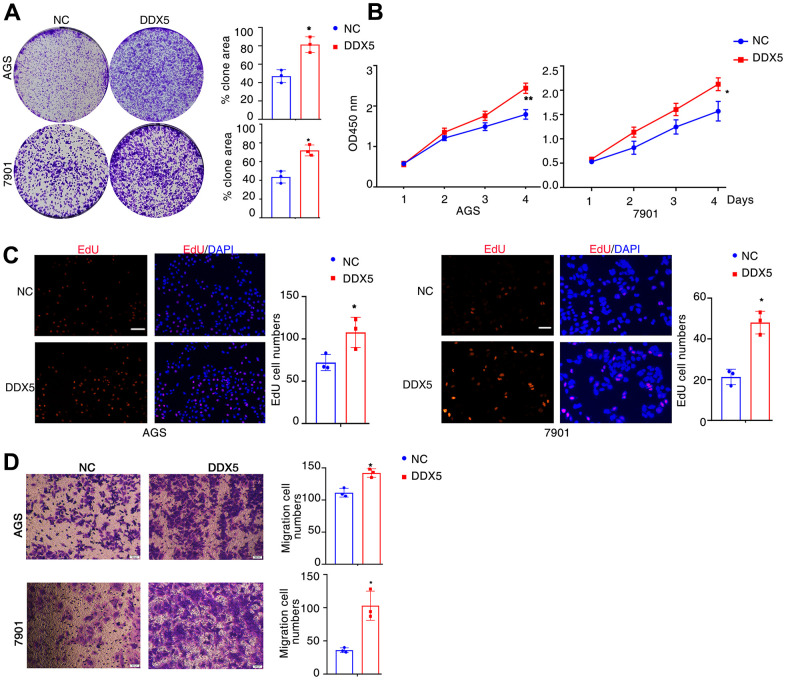
**DDX5 promotes cancer cell growth and migration.** (**A**) Colony formation of AGS and 7901 cells two weeks after transfection with the indicated vector. (**B**) CCK-8 assay of proliferation in AGS and 7901 cells following a time course of transfection with NC or DDX5. (**C**) EdU assay was conducted to test the effect of DDX5 expression on the proliferation of AGS and 7901 cells. Scale bar 100μm. (**D**) Transwell assay was conducted to test the effect of DDX5 expression on the migration of AGS and 7901 cells. Number of cells were counted and shown in the column graph on the right of the corresponding pictures. Data are mean ± SD of three independent experiments. * P < 0.05, ** P < 0.01. Scale bar 500μm.

Next, to assess the regulatory function of DDX5 in the migratory capacity of gastric tumour cell lines, DDX5 was transfected into AGS cells and 7901 cells using a DDX5 expression vector. DDX5 overexpression notably accelerated the proliferation and growth of gastric cancer cells as shown by tumor cell clonogenesis assay, EdU staining and CCK8 cell proliferation assay (Figure 2A–2C). For the purpose of testing the role of DDX5 in cell migration, we carried out transwell experiments in gastric cancer cells. DDX5 expression enhanced the migration capability in AGS and 7901 cell (Figure 2D). Together, it is suggested that DDX5 stimulates the growth and migration on gastric cancer cells *in vitro*.

### circRNAs are induced by DDX5

**Figure 3:**
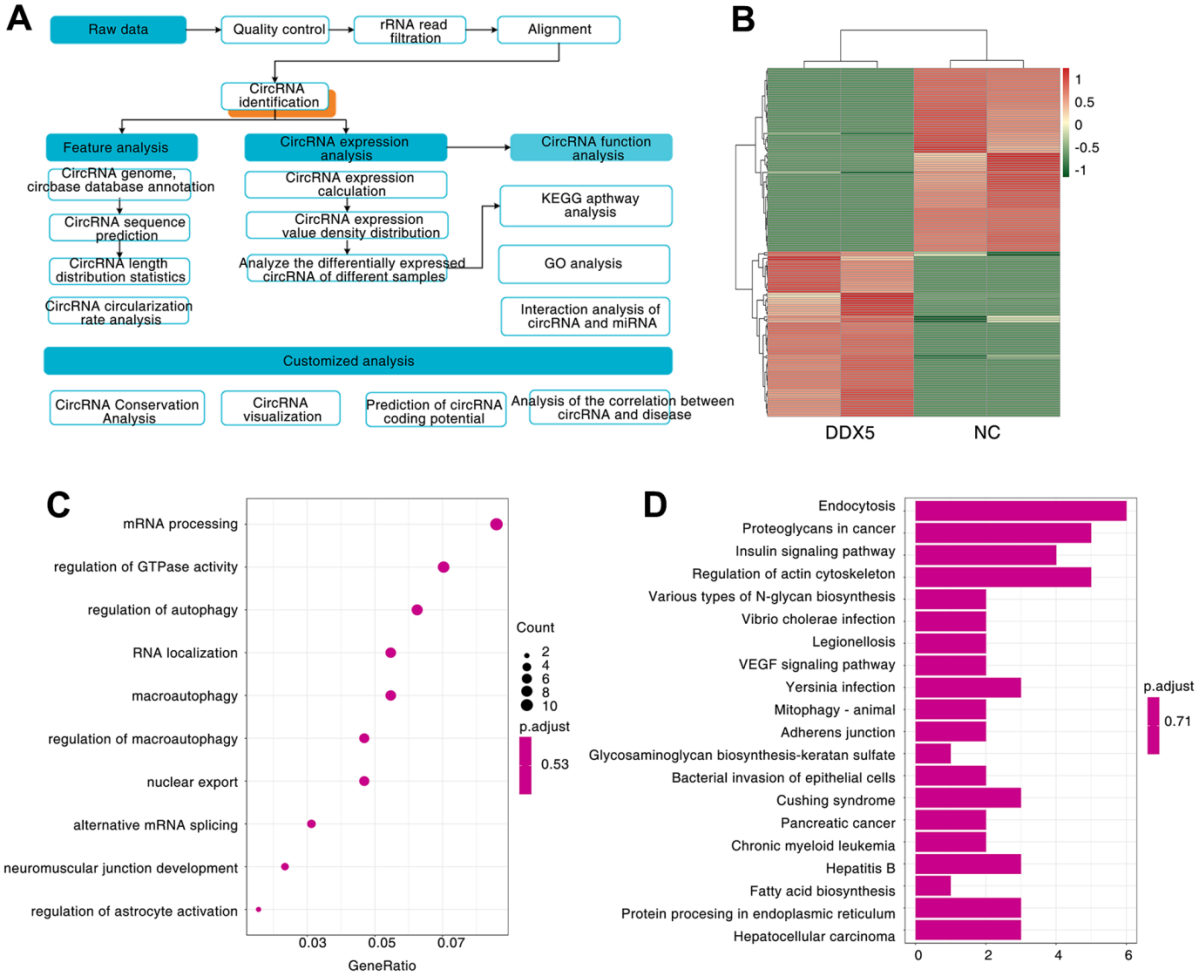
**A large number of circRNAs are induced by DDX5.** (**A**) Workflow for sequencing and analysis of DDX5-treated specimens. (**B**) Heatmap of differentially expressed circRNAs between NC and DDX5-overexpression AGS cells identified by circRNA-seq. (**C**) GO enrichment analysis was used to analyze the biological functions of the DDX5-regulated differentially expressed circRNAs. (**D**) KEGG pathway was used to analyze the pathways related to the DDX5-regulated differentially expressed circRNAs.

We know that circular RNA is obtained by covalently linking a downstream splicing donor to an upstream splicing acceptor, a backsplicing process catalyzed by the spliceosome [33]. DDX5, as a well-known splicing factor, it has been reported to regulate a large number of splicing events [34, 35]. Thus, we speculated whether DDX5 could promote the progression of gastric cancer cells via regulating the splicing formation of tumor-related circular RNAs. To identify whether the expression of DDX5 was linked to circular RNAs splicing processes, we transfected AGS cell with FLAG-DDX5 or FLAG-tagged empty vector (negative control, NC), and analyzed the differentially expressed circRNAs by Ribo zero rRNA removal kit and circRNA-seq (RNase R-treated RNA sequencing) (Figure 3A and Supplementary Figure 2). We analyzed the circRNA-seq data by CIRCexplorer2 and CIRI2software and 7587 circular RNAs were found in the control group and DDX5 overexpressed cells (Supplementary Table 1, Supporting Information). Next, we asked whether DDX5 could change the program of circRNAs in AGS cells via comparing confirmed circRNAs in DDX5-overexpressed cells to that of control cells. We found that DDX5 overexpression promoted or inhibited the expression of 72 and 80 circular RNAs, respectively (Supplementary Table 2, Supporting Information and Figure 3B). To reveal the potential biological roles of these DDX5-induced circRNAs, we carried out Gene Ontology (GO) and found that a lot of DDX5-induced circRNAs are components of mRNA processing (Figure 3C). Kyoto Encyclopedia of Genes and Genomes (KEGG) pathway enrichment analysis also revealed that a lot of DDX5-regulated circRNAs are mainly enriched in tumor-related signaling pathway, including VEGF and insulin signaling pathway (Figure 3D). These results suggested that DDX5 may regulate circRNA biogenesis.

### DDX5 alters circPHF14 expression

**Figure 4:**
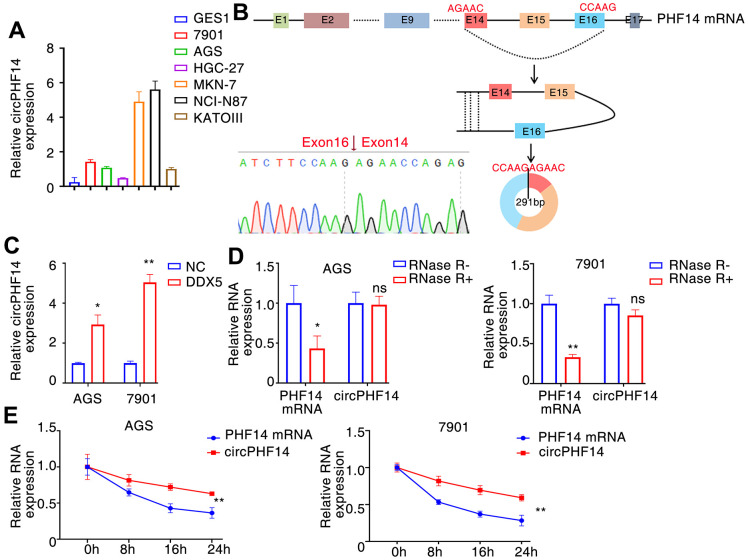
**DDX5 alters circPHF14 expression.** (**A**) Endogenous circPHF14 expression levels in multiple gastric cancer cells by RT-PCR. (**B**) We confirmed circPHF14 was formed by circularization of exons 14, 15, 16 in the PHF14 mRNA by Sanger sequencing, and also determined its genomic size and sequence. (**C**) RT-PCR was performed in AGS or 7901 cells transfected with DDX5, and then relative expression of circPHF14 was detected by qPCR. (**D**, **E**) The relative expression of circPHF14 and PHF14 mRNA in AGS or 7901 cells was detected by RT-PCR after RNase R treatment for 30 min (**D**) or actinomycin D treatment for 0 h, 8 h, 16 h, and 24 h (**E**). Data are mean ± SD of three independent experiments. * P < 0.05, ** P < 0.01, ns stands for no significance.

In order to identify novel circRNAs that plays a significant biological role in gastric cancer, particularly its growth and migration. In these DDX5-induced circular RNAs, we found that hsa_circ:chr7:11101418-11151094 has a very top expression change after DDX5 overexpression in AGS cells. Moreover, the expression of hsa_circ:chr7:11101418-11151094 in gastric cancer cell lines was significantly higher than that in normal immortalized GES1 cells (Figure 4A). RT-PCR and Sanger sequencing showed that hsa_circ:chr7:11101418-11151094 was formed by back-splicing of exons 14-16 of PHF14 gene (reference sequence: NR_033436.2). The total length of hsa_circ:chr7:11101418-11151094 is 291 nt, and its splicing sequence is consistent with that of hsa_circ_0079440 in circBase; Therefore, it is named circPHF14 (Figure 4B). We further detected the effect of DDX5 on the expression of circPHF14 in gastric cancer cells by qRT–PCR. The results showed that the expression of circPHF14 changed continuously with the increase of DDX5 level in AGS and 7901 cells (Figure 4C). We treated gastric cancer cells with actinomycin D or RNase R to inhibit intracellular RNA transcription. The qPCR experiment showed that the circPHF14 was more stable than the linear mRNA of the PHF14 gene (Figure 4D, 4E).

We also found a significant co-localization between DDX5 and circPNF14 using immunofluorescence experiments (Supplementary Figure 3).

### Silencing circPHF14 reverses the DDX5-induced tumor-promoting effects in gastric cancer cells

**Figure 5:**
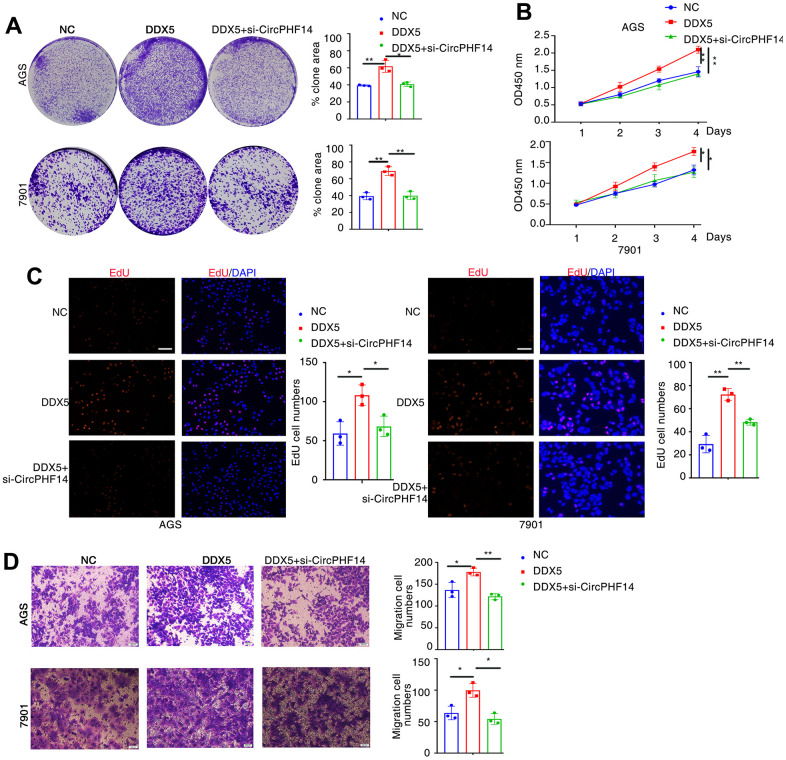
**DDX5 promotes gastric cancer cells growth and migration by regulating circPHF14.** (**A**) AGS or 7901 cells transfected with control siRNA or siRNA specifically targeting circPHF14 (si-circPHF14) in the presence DDX5 were subjected to colony formation assay. (**B**) CCK-8 assay of proliferation in AGS and 7901 cells following a time course of transfection with NC or DDX5 or DDX5 and si-circPHF14. (**C**) EdU assay was conducted to test the effect of DDX5 and circPHF14 on the proliferation of AGS and 7901 cells. Scale bar 100μm. (**D**) A migration rescue experiment verified the effect of DDX5 and circPHF14 on the migration ability of AGS and 7901 cells. Data are mean ± SD of three independent experiments. * P < 0.05, ** P < 0.01. Scale bar 500μm.

To prove that DDX5 promotes the development of gastric cancer cells, at least in part by regulating the expression of circPHF14, it is important to confirm whether inhibition of circPHF14 can reverse the effect of DDX5 on gastric cancer cells. Thus, rescue assays were performed via co-transfecting DDX5 expression vector and si-circPHF14 or si-NC (Supplementary Figure 4). Knockdown of both circPHF14 could partly rescue the excessive proliferation abilities of overexpressed DDX5 gastric cancer cell lines compared with the negative control group by plate colony formation, CCK-8 and EdU staining assays (Figure 5A–5C). Additionally transwell migration assay shown that the promotion of cell motility ability was reversed via the inhibition of circPHF14 expression (Figure 5D).

### DDX5 promote gastric cancer cells progression *in vivo* via circPHF14

**Figure 6:**
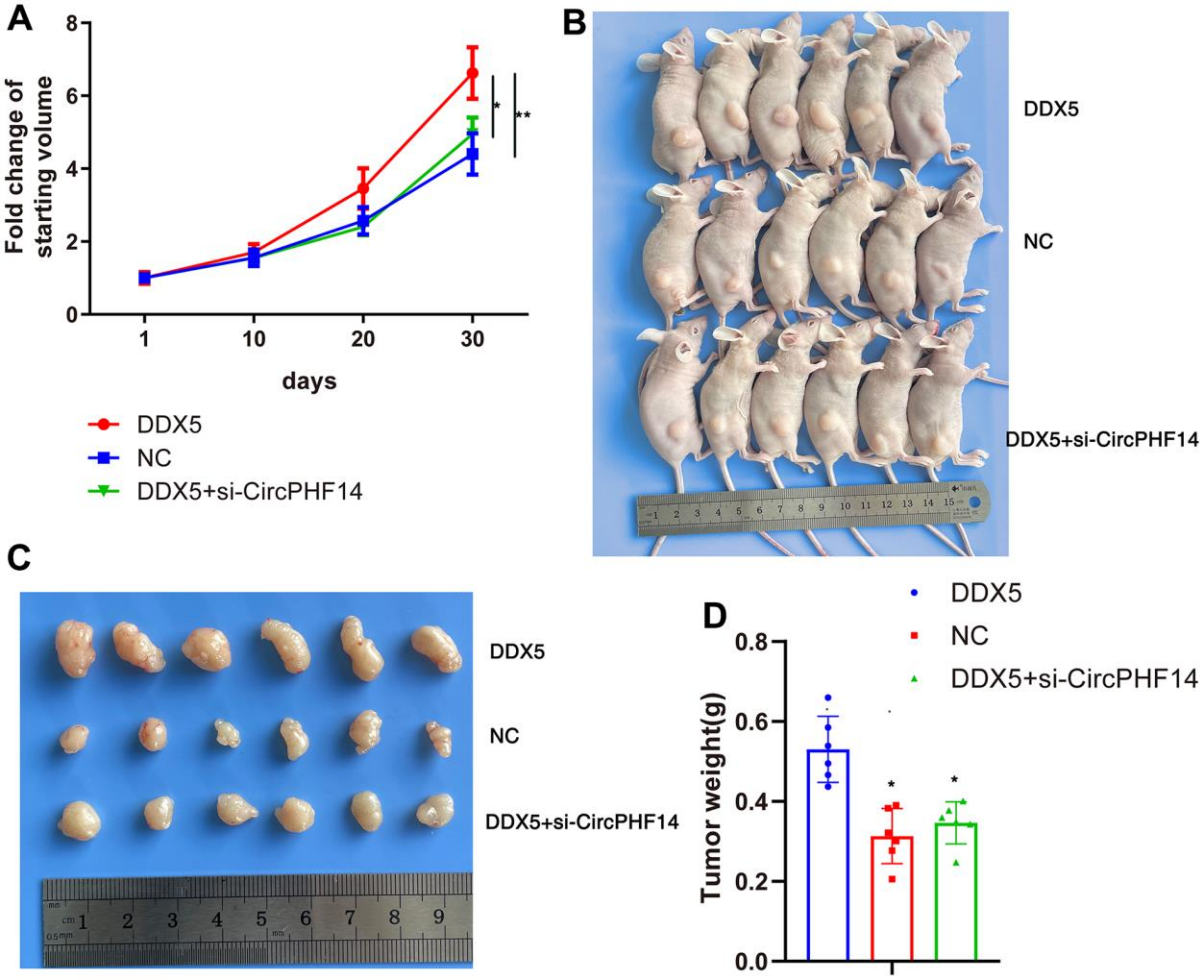
**DDX5 promote gastric cancer cells progression *in vivo* via circPHF14.** (**A**) Tumor growth curves of AGS xenografts in nude mice. Group 1, 2, 3 mice were injected with DDX5, NC, DDX5 and si-circPHF14 cells, respectively. (**B**–**D**) Xenograft tumors collected on day 30 post subcutaneous implantation.

To determine whether DDX5 and circPHF14 an extremely significant function in the development of gastric cancer cells *in vivo*, we established a xenograft nude tumor model. Stable transfection of AGS gastric cancer cells with NC or co-transfected with DDX5 and sh-NC or co-transfected with DDX5 and sh-circPHF14, and injected separately into the nude mice. As shown in Figure 6A, 6B, the DDX5 overexpressed group had higher proliferation rate than the negative control group, whereas cells silenced for circPHF14 partially reversed the inhibition in proliferation. In addition, the volume of AGS-derived tumors was inhibited by reducing the expression of circPHF14 *in vivo* (Figure 6C, 6D). These findings suggest that DDX5 may play a key role in accelerating the growth of gastric cancer cells through circPHF14 *in vivo*.

## DISCUSSION

DDX5 was originally identified as an RNA helicase that is involved in almost all aspects of RNA process, including pre-mRNA splicing, ribosomal biogenesis and miRNA maturation [36]. Certain previous works had illustrated that DDX5 is frequently overexpressed in many malignancies and that its dysregulated expression is responsible for tumorigenesis and progression [37, 38]. Moreover, DDX5 binds to β-catenin and promotes the expression of several oncogenes such as cell cycle protein D1, c-Myc and c-jun [14]. DDX5 is frequently amplified in breast cancer and is closely coupled with the proliferation of cancer cells [39]. In gliomas, DDX5 can interact with p50 to enhance its accumulation and transcriptional activity in the nucleus, leading to promoting tumor growth [40]. In gastric cancer, DDX5 contributes to the evolution of gastric cancer cells mediating the mTOR signaling pathway activation [15]. In our study, we also found that there is an upregulation of DDX5 expression in cancerous tissues compared to paraneoplastic tissues, and that high DDX5 expression is closely associated with the prognosis of patients with gastric tumours.

Recent years, with the development of high-throughput sequencing technology and molecular biology, increasing research began to focus on the function and mechanism of circRNA. There have been a large number of research reports on the impact of circRNA on the development of cancer [41]. It is universally known that stomach cancer is one of the most common malignant tumors in the world. Some studies have reported that circular RNA plays an extremely critical role in the development and progression of gastric carcinoma. For instance, Guan et al. reported that circRNA promoted gastric cancer cell growth and migration via reducing the transcriptional activity of miR-637 [42]. Li et al. revealed that circRNAs enhanced gastric cancer cell growth by regulating the miR-503/CACUL1 signaling [43]. The expression of mRNA, miRNAs and lncRNAs induced by DDX5 has been proved to plays an extremely critical role in the beginning and advancement of cancer. However, whether DDX5 can induce the expression of circRNAs and, if so, what role these circRNAs play in DDX5-positive gastric tumor is unclear. In this research, through CircRNA-seq, we found that DDX5 induced the expression of a lot of circRNAs in gastric cancer cells, such as circPHF14. DDX5-induced circRNAs may have the function of enhancing the development of gastric cancer cells, which may offer new targets for subsequent diagnosis and treatment of DDX5-positive gastric tumor.

Circular RNA has been found to regulate a large number of biological processes, including gene splicing, miRNA sponge and transcriptional regulation, etc. [41, 44]. One of the most classical mechanisms of noncoding RNA (ncRNAs) is miRNA sponge effect, including circular RNAs [45, 46]. For example, Wang et al. showed that circPGR, located in the cytoplasm, regulates the expression of multiple cell cycle genes through competing endogenous RNA to sponge miR-301a-5p [47]. Chen et al. revealed also that circFNTA can promote the expression of FNTA by competing with microRNA miR-370-3p, thereby activating KRAS signaling, promoting the invasion and cisplatin resistance of bladder cancer cell [48]. In our research, whether DDX5-induced circPHF14 can also play a role by competes with the microRNA as a sponge requires further research.

In conclusion, our data has shown enhanced ddx5 expression in gastric cancer tissues and identified that ddx5 can facilitate gastric cancer cell growth and migration *in vitro*. Moreover, we generated RNase R-treated RNA-seq data in AGS cells overexpressing DDX5 and identified a wealth of DDX5-induced circRNAs, in which circPHF14 enhances DDX5-positive gastric cancer cell growth and tumorigenesis *in vivo* and *in vitro*. Summarize all the above research, DDX5 and these DDX5-induced circRNAs play a key role in the occurrence and development of gastric cancer, and might be a potential target for gastric cancer treatment.

## Supplementary Material

Supplementary Figures

Supplementary Table 1

Supplementary Table 2
