# Supplementary material for: RNA helicase DDX5-induced circPHF14 promotes gastric cancer cell progression
Source: Aging (Albany NY). 2023 Mar 30;15(7):2525–40. doi: 10.18632/aging.204623 (PMC10120908; doi:10.18632/aging.204623)
Supplement: Supplementary Figures [file aging-15-204623-s001.pdf]

SUPPLEMENTARY FIGURES

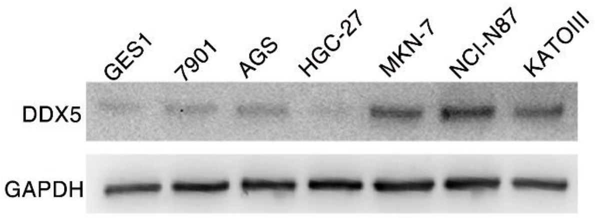

Supplementary Figure 1. Western blot assay detects the protein expression level of DDX5 in normal cells and multiple gastric cancer cell lines.

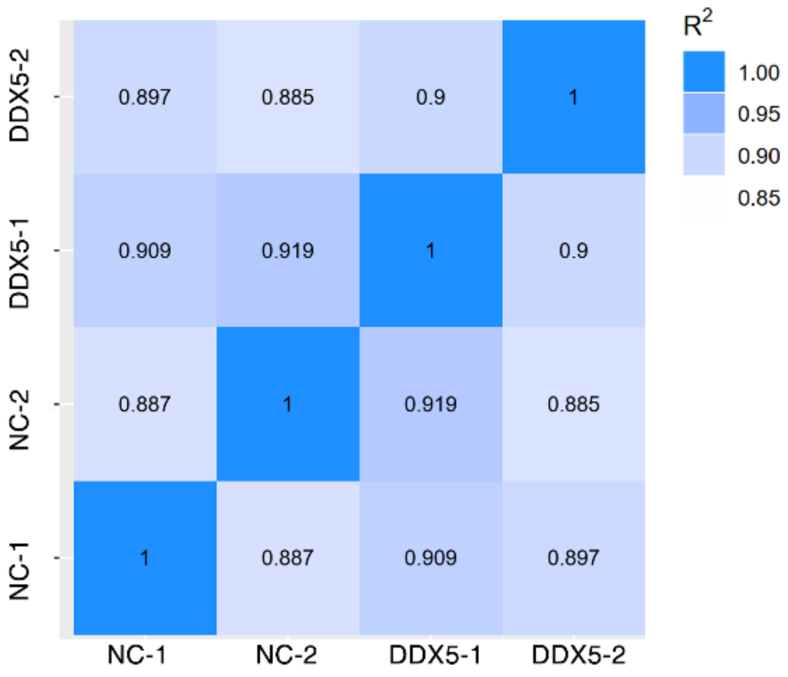

Supplementary Figure 2. Correlation analysis between circRNA-seq samples.

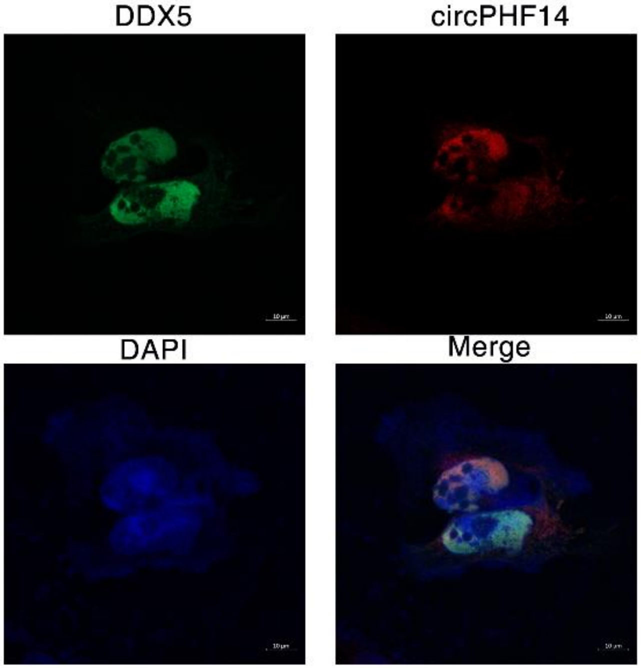

Supplementary Figure 3. Immunofluorescence assay to detect the localization of DDX5 and circPHF14.

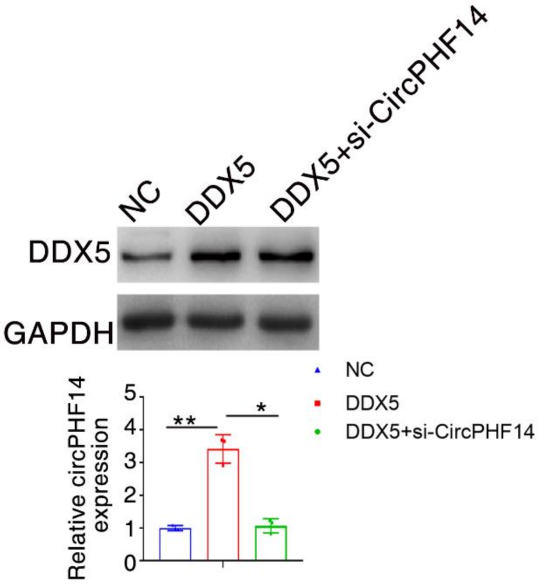

Supplementary Figure 4. Western blot and qPCR experiments to detect the expression levels of DDX5 and circPHF14 after treatment as shown.
